# Family structure and posttraumatic stress reactions: a longitudinal study using multilevel analyses

**DOI:** 10.1186/1471-244X-11-195

**Published:** 2011-12-15

**Authors:** Egil Nygaard, Tore Wentzel-Larsen, Ajmal Hussain, Trond Heir

**Affiliations:** 1 Norwegian Centre for Violence and Traumatic Stress Studies, Kirkeveien 166, Building 48, 0407 Oslo, Norway; 2 Centre for Child and Adolescent Mental Health, Eastern and Southern Norway, Gullhaug Torg 4B, 0484 Oslo, Norway

**Keywords:** family structure, multilevel analyses, posttraumatic stress reactions, PTSD, tsunami

## Abstract

**Background:**

There is limited research on the relevance of family structures to the development and maintenance of posttraumatic stress following disasters. We longitudinally studied the effects of marital and parental statuses on posttraumatic stress reactions after the 2004 Southeast Asian tsunami and whether persons in the same households had more shared stress reactions than others.

**Method:**

The study included a tourist population of 641 Norwegian adult citizens, many of them from families with children. We measured posttraumatic stress symptoms with the Impact of Event Scale-Revised at 6 months and 2 years post-disaster. Analyses included multilevel methods with mixed effects models.

**Results:**

Results showed that neither marital nor parental status was significantly related to posttraumatic stress. At both assessments, adults living in the same household reported levels of posttraumatic stress that were more similar to one another than adults who were not living together. Between households, disaster experiences were closely related to the variance in posttraumatic stress symptom levels at both assessments. Within households, however, disaster experiences were less related to the variance in symptom level at 2 years than at 6 months.

**Conclusions:**

These results indicate that adult household members may influence one another's posttraumatic stress reactions as well as their interpretations of the disaster experiences over time. Our findings suggest that multilevel methods may provide important information about family processes after disasters.

## Background

There has been increasing interest in the relevance of family factors to the development of posttraumatic stress disorder (PTSD). In particular, family functioning and intrafamily support have been considered important [1-7]. Singles may receive less family support than married persons due to lack of partner [8]. However, the impact of marital status on posttraumatic stress is ambiguous. Some studies have found married individuals to have less posttraumatic stress reactions than unmarried individuals [4,9,10], while others have found the opposite effect [11] or no relationship between the variables [12-14]. Whereas one study found that divorced, separated, or widowed adults are at higher risk for PTSD than people who are presently married [15], another study found that this risk disappeared when controlling for other sociodemographic factors and trauma categories [16].

Few post-disaster studies have examined the effect of parental status on the development of PTSD. Parenthood may influence the risk of developing posttraumatic stress reactions through processes occurring both in the acute disaster situation [17] and post-disaster. Such an effect would be in accordance with classical developmental theories of bidirectional processes between parents and children [18,19] as well as with more contemporary developmental theories [20,21]. However, whereas several studies have found parental factors to be related to children's development of PTSD after disasters [22-25], relatively few longitudinal studies have investigated how parents are influenced by their children's level of posttraumatic stress reactions, and these studies have yielded discrepant results [26-29]. Furthermore, few studies have investigated whether having children relates to levels of posttraumatic stress. Studies have found that parents had higher levels of posttraumatic stress than nonparents after the Chernobyl disaster [30], after floods [8,14], and after the 9/11 attacks [31]. The effect was particularly pronounced for single parents impacted by the 9/11 attacks [32]. However, other work suggest that parenthood or being in the company of children were not risk factors for posttraumatic stress reactions after the 2004 tsunami [10,12].

An alternative method of investigating the relevance of family factors to posttraumatic stress reactions is by examining similarities in reactions within the family. We found only three studies that looked at the similarities of couples' reactions to disasters [14,33,34]. All three studies found general mental health or depression to be more similar within couples than for non-related adults but did not measure specific posttraumatic stress reactions. Two other studies found child siblings' posttraumatic stress reactions not to be more similar than other children's reactions [35,36], thus it is unclear if family members do actually have more similar reactions after disaster than other disaster victims. If more than one person from a family is included in a study, the participants' responses are not independent of each other. Such grouping effects may influence results [37]; therefore, it has been suggested that disaster research should take grouping into account [38]. Multilevel analysis, including mixed effect models, is such a statistical method. It takes into account that some participants come from same subgroup, and thus for example analyze both the variability between individuals and between families [37]. However, very few disaster studies have taken into account the mutual experiences and shared reactions of families when analyzing predictors of posttraumatic stress reactions [10,14,39,40]. Some resolve the problem by investigating only one participant from each household [7,31] or by using sampling weights to correct for selection bias related to number of household members [32]. Others make no adjustments to account for participants from the same household [12,33]. Thus, the question remains to what extent adult participants living in same household do have more similar reactions than other participants, and thereby are not independent observations, and how such possible grouping effects should be taken into account. This is important because the assumption of independent observations is one of the basic assumptions in standard statistical analyses.

### Present study

This longitudinal study investigated posttraumatic stress reactions in Norwegian adults who experienced the tsunami as tourists in Southeast Asia on December 26, 2004. To our knowledge, this is the first longitudinal study of posttraumatic stress reactions to account for the multilevel effect of mutual households or families within the sample. We aimed to examine the relevance of family structures for adults' risk of posttraumatic stress reactions using two strategies: (1) by analyzing family structures as predictors for posttraumatic stress reactions and (2) by investigating possible similarities in reactions within families. The specific aims of the study were as follows:

To investigate differences in posttraumatic stress reactions between married participants and non-married participants

To investigate differences in posttraumatic stress reactions between parents and adults without children

To investigate, via multilevel analyses, whether adults within shared households had more similar posttraumatic stress reactions than adults from different households

## Methods

### Procedure

Shortly after the 2004 tsunami in Southeast Asia, Norwegian nationals who were evacuated from the disaster-stricken area were registered upon arrival in Norway. A postal questionnaire was sent to all registered persons 18 years or older (*N *= 2468) at 6 (T_1_) and 24 (T_2_) months post-tsunami. The questionnaire at T_1 _included questions concerning exposure, posttraumatic stress reactions, marital and parental status, and other background variables [41]. The questionnaire at T_2 _included questions about posttraumatic stress reactions [42]. Participants with the same address were assumed to be living in a common household. The study was approved by the Norwegian Social Science Data Services and The Regional Committee for Medical Research Ethics.

### Participants

While 868 and 1170 responded at T_1 _or T_2 _respectively, we received questionnaires for both T_1 _and T_2 _from 657 respondents. Five of these were excluded due to high levels of missing data on measures of posttraumatic stress reactions, and eleven more were excluded due to missing addresses. Therefore, the final sample included 641 participants.

At T_1_, 61.8% of the 641 participants had more than 12 years of education, and 75.5% were employed. There were multiple participants from the same household in 221 cases (34.5%). A total of 48.4% of the participants reported that they had traveled with a spouse or cohabitating partner. A total of 247 (38.5%) participants reported having children under the age of 18 years at T_1_; 240 participants (40.7%) reported to have responsibility for a child at time of the disaster, including 25 (4.2%) who had sole responsibility; and 310 (48.4%) reported to have traveled together with their own child, stepchild or foster child. At T_1_, 70.5% were married or cohabitating, 9.4% were no longer married and 20.1% were single. A total of 50 participants changed marital status from T_1 _to T_2_, 27 of which were no longer married or cohabitating, and 23 participants became married/cohabiter. More descriptive information about the participants is included in Table 1.

**Table 1:** Descriptive statistics of the major study variables (N = 641)

*Variable*	*n (%)/M (SD)*
Number of participants from household at T_1_	
One	420 (65.5%)
Two	196 (30.6%)
Three	21 (3.3%)
Four	4 (0.6%)
Mean age at time of tsunami (*SD*)	43.4 (12.9)
Sex	
Men	288 (44.9%)
Women	353 (55.1%)
Marital status at T_1_	
Married or cohabitating	434 (70.5%)
Divorced, separated, or widowed	58 (9.4%)
Single	124 (20.1%)
Missing	25
Had children under 18 years of age at T_1_	
No	394 (61.5%)
Yes	247 (38.5%)
Witnessed abandoned children	
No	415 (70.2%)
Yes	176 (29.8%)
Missing	50
Witnessed multiple dead bodies	
No	463 (78.9%)
Yes	124 (21.1%)
Missing	54
Caught, touched or chased by waves	
No	404 (64.0%)
Yes	227 (36.0%)
Missing	10
Death of family member or friend	
No	585 (91.3%)
Yes	56 (8.7%)
Mean immediate response of fear during tsunami (*SD*)	2.5 (1.4)^a^
Mean immediate response of helplessness during tsunami (*SD*)	2.6 (1.4)^b^
Mean posttraumatic stress reactions at T_1 _(*SD*)	1.1 (0.8)
Mean posttraumatic stress reactions at T_2 _(*SD*)	1.0 (0.8)

^a ^*n *= 596. ^b ^*n *= 597.

Non-responders at T_1 _were more likely than responders to have resided in less severely affected locations in Southeast Asia [41] and were more often men; however, they were similar in age to responders [43]. The most frequently reported reasons for not participating were lack of interest or time, followed by lack of relevant experiences [44]. The final sample did not differ from responders who were excluded from the analyses in family features (marital status, proportion who had children at T_1_, or proportion of participants from same household) or posttraumatic stress reactions at T_1_. However, the excluded responders reported a lower average level of posttraumatic stress reactions at T_2 _than the analyzed sample (*M(SD)*_excluded _= 0.85 (0.82), *M*_included _= 1.05 (0.83), *t*(1396) = 2.89, *p *= .004).

### Measures

#### Exposure and immediate response to the disaster

Based on earlier work [45], questions regarding a broad spectrum of tsunami experiences were assessed in the questionnaire 6-months post-tsunami. Based on earlier evaluations of the exposure experiences as risk factors [41], four questions were included in the present study to measure exposure: whether a participant had witnessed multiple dead bodies, had witnessed abandoned children, had been caught, touched or chased by waves, or had experienced the death of a family member or friend. Each question was answered *no *(0) or *yes *(1). Two questions were used to assess immediate subjective response to the disaster: fear, and feelings of helplessness, with both items rated on a five-point scale (0 = *not at all*, 1 = *little*, 2 = *moderate*, 3 = *intense*, 4 = *extreme*). These two items represented immediate response to the disaster, corresponding to the A2 criterion for PTSD from the DSM-IV [46].

#### Posttraumatic stress reactions

The Impact of Event Scale - Revised (IES-R) [47] was included at both assessments to measure the level of posttraumatic stress reactions during the previous two weeks. The IES-R includes 22 items with five response alternatives (0 = *not at all*, 1 = *little*, 2 = *moderately*, 3 = *quite a bit*, 4 = *extremely*). Total mean scores were based on all items. The psychometric properties of the IES-R have been extensively evaluated and deemed acceptable, with internal consistency within subscales reported to be between .81 and .91, test-retest reliability to be between .52 and .86, and correlation with other measures of posttraumatic stress reactions to be between .53 and .57 [48]. Similar acceptable measures or reliability have been found in a Norwegian non-clinical sample [49]. The internal consistency was high in the present sample (Cronbach's α = .96 and .95 at T_1 _and T_2_, respectively).

### Data analysis

We excluded participants who were missing more than four replies to questions about posttraumatic stress reactions. For the remaining participants, missing values for these variables were replaced using expectation maximization algorithms (EM algorithms), which took into account a participant's scores on items within the same symptom cluster, the scores of the other respondents, and the correlations between items [50]. Dropout analyses were done using χ^2^-tests for categorical data and student t-tests for continuous data.

Chi-square tests were used for bivariate analyses of grouped variables. The effects of marital status and parental status on posttraumatic stress reactions were first tested with univariate mixed effects models adjusted for exposure and immediate subjective distress during the disaster. The effect of single parenthood was tested with a mixed effects model with random effects for differences between household and for individuals within household. There were no random effects regressors. Fixed effects regressors included both marital status and parental status, and also adjustment for the six questions concerning exposure and immediate subjective distress during the disaster. Mixed effects models were also used to analyze whether household members reported more similar posttraumatic stress reactions than participants from different households. The effect of household was tested with two mixed effects models, first an unadjusted model without predictors and next with a model adjusted for exposure and immediate subjective distress during the disaster. In this way, all models controlled for participants who lived in the same household. This multilevel approach means that the regression model has error terms at two levels, the individual level and the household level.

Similarity between household members is presented by intra-class correlations (ICC) computed from the random effects between and within household in the mixed effects models. ICC was calculated by dividing unexplained variance between households by the sum of unexplained variance between households and between individuals within same household. ICC can vary between 0 and 1. An ICC close to 0 indicates that household members are no more similar than other participants, whereas an ICC of 1 indicates that household members have identical responses. Confidence intervals for ICC were based on parametric bootstrapping and computed as bootstrap percentile intervals using 10,000 bootstrap replications. Bootstrapping is a general procedure that e.g. makes it possible to compute confidence intervals in cases where other methods are not easily available [51].

All tests were two-tailed, with a significance level of *p *≤ .05. Statistical analyses were performed using PASW Statistics, version 18, and R, version 2.10.1, with packages nlme and boot.

## Results

### Marital status and parenthood

Marital status at T_1 _was not related to the level of posttraumatic stress reactions at T_1 _or at T_2_. The mean values (*SD*) of IES-R at T_1 _and T_2_, respectively, were 1.1 (0.8) and 1.0 (0.8) for married/cohabiters, 1.1 (0.8) and 1.1 (1.0) for single persons who had been previously married, and 1.1 (0.9) and 1.0 (0.8) for single persons who had not been previously married, corresponding to an average response close to *little *on the 0-4 scale (*F*(2, 515.2) = 0.02, *p *= .98 at T_1 _and *F*(2, 512.5) = 1.74, *p *= .18 at T_2_).

Furthermore, parents at T_1 _did not differ from non-parents in their level of posttraumatic stress reactions at T_1 _or at T_2_. The mean values (*SD*) of IES-R at T_1 _and T_2_, respectively, were 1.2 (0.9) and 1.1 (0.8) for non-parents and 1.1 (0.8) and 1.0 (0.8) for parents (*t*(503.8) = 1.77, *p *= .08, *b*(95% CI) = 0.10 (-0.01, 0.22) at T_1 _and *t*(469.4) = 0.91, *p *= .36, *b*(95% CI) = -0.06 (-0.07, 0.18) at T_2_).

To examine whether posttraumatic stress reactions differed between single parents and couples with children, marital status (single, divorced, separated, or widowed versus couple), parenthood (having a child versus not having a child), and the interaction effect between marital status and parenthood, were entered simultaneously into mixed effects models. No significant main effects or interaction effects on posttraumatic stress reactions were found at either of the two study times.

Further analyses were conducted to investigate whether parents and non-parents differed in their exposure or immediate emotional reactions during the tsunami. Compared to non-parents, parents did not witness more abandoned children, were not taken more often by the waves, and did not feel more helplessness during the tsunami. However, parents were less likely to have witnessed multiple dead bodies (χ^2^(1, *N *= 587) = 5.38, *p *= .02), were more likely to have lost family or friends in the tsunami (χ^2^(1, *N *= 641) = 8.97, *p *= .003), and felt more fear during the tsunami (t(505.2) = 2.39, p = .02, *b*(95% CI) = 0.28 (0.05, 0.52)).

### Mutual household

At both time points, adults from the same household reported more similar levels of posttraumatic stress reactions than adults from different households. The unadjusted intra-class correlation (ICC) for posttraumatic stress reactions in the mixed effects model was .53 at T_1 _and .47 at T_2 _(Table 2). The confidence intervals for ICC at both times were sufficiently far from zero to indicate a considerable effect of mutual household. To examine whether similarities between members of the same household could be due to a greater number of shared experiences during the tsunami, we performed mixed effects models adjusted for the six questions concerning exposure and immediate emotional responses. Similar results were found, with ICC of .56 and .35 at T_1 _and T_2_, respectively (Table 2).

**Table 2:** Intra-class correlations for posttraumatic stress reactions, with and without adjustments for predictors at 6 (T_1_) and 24 (T_2_) months post-tsunami, and differences between these times

	*Without adjusting for predictors (95% CI)^a^*			*Adjusting for predictors (95% CI)^b^*		
	**T**_**1**_	**T**_**2**_	**Difference (T_2_-T_1_)**	**T**_**1**_	**T**_**2**_	**Difference (T_2_-T_1_)**
Unexplained variance between households	0.38	0.33	-0.05	0.23	0.17	-0.06
Unexplained variance between individuals within households	0.33	0.37	0.04	0.18	0.32	0.14
Intra-class correlation	.53 (.36, .62)	.47 (.45, .68)	-.06 (-.24, .10)	.56 (.42, .69)	.35 (.17, .52)	-.21 (-.43, .005)
Change in unexplained variance between households when including predictors				-44.8%	-48.4%	
Change in unexplained variance between individuals within households when including predictors				-36.0%	-9.0%	
Total change in unexplained variance when including predictors				-41.3%	-28.2%	

Adjustment for mutual household is done by mixed method with mutual household as intercept. Dependent variable is mean posttraumatic stress reactions (IES). Intra-class correlation (ICC) is defined as the proportion of unexplained variance that is between groups (possible range 0-1). It is calculated as unexplained variance between households divided by the sum of unexplained variance between and within households. Predictors controlled for include witnessed abandoned children, witnessed multiple dead bodies, caught or chased by waves, death of family member or friend, immediate response of fear, and immediate response of helplessness.

Difference (T_2_-T_1_) is the difference between the two assessment points in unexplained variance and intra-class correlation.

Change in variance in analyses adjusted for predictors is the change in unexplained variance when taking into account the predictors in percentage of unexplained variance before taking into account any predictors. The percentages are different from what can be calculated from the first part of the table because the change in unexplained variance is based on models excluding participants with missing data on predictor variables (N = 550).

^a ^N = 641. ^b ^N = 550.

The decrease in unadjusted and adjusted ICC from T_1 _to T_2 _was not significant. However, the decrease in adjusted ICC was close to significant (Table 2). This decrease was related to changes in how much tsunami experiences explained variance in posttraumatic stress reactions between individuals within families. Taking into account the disaster experiences, the unexplained variances at T_1 _were reduced both at the individual level (36.0%) and at the household level (44.8%). At T_2_, unexplained variance between households had decreased (48.4%), whereas unexplained variance between individuals within the same household had decreased less when taking into account the disaster experiences (9.0%) (Table 2). Thus, tsunami experiences were still related to posttraumatic stress reactions of families at T_2_, but not as much to the reactions of individuals within families.

## Discussion

In the present study, neither marital status nor parental status was related to the level of posttraumatic stress. Adults living in the same household had more similar levels of posttraumatic stress than adults not living together. The association between household members with regard to posttraumatic stress did not change from T_1 _to T_2_. Disaster experiences were associated with posttraumatic stress of individuals within families at T_1_, but there was almost no such association at T_2_. Thus, the impact of each family member's original disaster experiences on the level of posttraumatic stress decreased over time.

Neither single nor married parents had higher levels of posttraumatic stress reactions than adults without children. This is in accordance with findings in a similar study of Swedish tourists during the tsunami [10] and a study of Sri Lankan adults who were displaced after the tsunami [12]. However, other studies have found parenthood to be related to higher levels of reactions after a nuclear accident [30], floods [8,14], and the 9/11 attack [31]. One possible reason for the discrepant findings is that our study, like the Swedish tsunami study, examined disaster survivors who were repatriated to stable home societies; thus, parents had fewer post-tsunami worries about their children's wellbeing and future. The fact that parents experienced both more fear than nonparents and were less likely to have witnessed dead bodies may have influenced our results as well. While the parents may have been more anxious because they worried about the wellbeing of their children, they may also have protected their children and thus also themselves from witness experiences. Thus, the findings indicate that it is possible that having children may both be related to factors enhancing and factors decreasing the risk of posttraumatic stress reactions [17], with such effects possibly nullifying each other.

Marital status was not related to an elevated or reduced level of posttraumatic stress reactions. This was surprising as singles may get less family support, and social support has been found to be a major protective factor against posttraumatic stress reactions [52,53]. However, adults living in the same household had more similar levels of posttraumatic stress reactions than adults not living in the same household, with ICC indicating that approximately half of the variation in posttraumatic stress reactions was related to differences between adults within the same household. This is consistent with theories indicating that humans in relationships influence each other and often have converging interpretations of mutual experiences [54-56]. In most natural disasters, all members of a family are exposed, and in most instances, the members of the family live together after the disaster. Therefore, not only do family members have more resemblance in their disaster experiences than unrelated people, but they will also influence each other's coping strategies, recollections, interpretations and post-disaster reactions. However, child siblings have been found to have unrelated posttraumatic stress reactions, both in the same population as the present sample and after common disaster experiences during an earthquake [35,36]. It is unclear why adult family members' reactions should be more similar than the reactions of child siblings. The difference between couples and child siblings may be age related and/or role related. Children are developmentally different from adults in how they are aware of their surroundings and their internal experiences [57]. It is also possible that couples communicate more and listen more to each other about the traumatic event and later reactions than siblings because of their cooperating role, especially their parental role. Whereas siblings may actively disagree, parents may seek cooperation and converging interpretation with their partner and co-caregiver.

We did not find any increase in household concordance of posttraumatic stress reactions from T_1 _to T_2_. This indicates that most of the family converging processes happened within six months of the disaster. However, the experiences during the disaster were less related to the posttraumatic stress reactions of the individuals within households at follow up than at the first assessment. These results indicate that family members influence each other's interpretations of the disaster over time. Thus, individual differences in interpretation of and reaction to the disaster diminish over time. The results thus indicate that over time, an individual's posttraumatic stress reactions may be influenced more by family members' interpretations and memories of the trauma than by actual exposure during the disaster.

### Methodological considerations

This study had some methodological advantages. Almost all Norwegians who were tourists in the disaster area were invited to participate, reducing sample selection bias. The participants experienced a single, easily identifiable trauma and were largely protected from secondary adversities because they were able to quickly return to unaffected home communities. The tsunami-related processes between the persons in the household should therefore be less influenced by processes outside the household.

There was a relatively low response rate. However, due to the directionality of the dropout bias, the included participants seem to represent most of the heavily exposed Norwegian tourists in the tsunami-stricken areas [44].

The information was gathered by the use of postal questionnaires. Thus, participants from same household may have interacted during the filling out process. This may have influenced the results.

This study is limited to symptoms of posttraumatic stress. Other consequences of the disaster exposure, such as depression or functional impairment, were not analyzed or discussed. Children's levels of posttraumatic stress reactions were not controlled for in the present analyses, even though parent's and children's posttraumatic stress reactions are often related [58]. Other traumatic experiences were not taken into account [59].

The present article has evaluated marital status at first assessment as a risk factor for later posttraumatic stress reactions. Additional analyses indicated that change in marital status was not related to level of posttraumatic stress reactions (results not shown). Additional analyses (not shown) did also find that neither traveling with children nor having responsibility for children during the tsunami were related to level of posttraumatic stress reactions.

## Conclusions

Adults living in the same household reported similar posttraumatic stress reactions. In addition, family members' interpretations of the disaster seemed to merge over time. This may be positive if the family moves in a favorable direction, but it indicates that for individuals who are not improving from posttraumatic stress reactions, it is important to investigate how their family interprets and perhaps contributes to the non-improving mental health. This study emphasizes the importance of a family-centered care that takes on an ecological grounded perspective when treating adults with posttraumatic stress reactions after common disaster experiences.

Methods of analysis that take into account the grouping of stress reactions within households provided valuable information about possible family processes. The study thus supports the importance of taking group levels into account when analyzing and discussing findings from studies including more than one participant from same household [38].

## Competing interests

The authors declare that they have no competing interests.

## Authors' contributions

EN performed the statistical analysis and drafted the manuscript. TWL performed the statistical analysis and was involved in the revision of the manuscript. AH collected data and revised the manuscript. TH participated in the conception, design, and revision of the manuscript and was the general supervisor for the project. All authors read and approved the final manuscript.

## Pre-publication history

The pre-publication history for this paper can be accessed here:

http://www.biomedcentral.com/1471-244X/11/195/prepub

## References

[B1] McFarlane AC Family functioning and overprotection following a natural disaster: The longitudinal effects of post-traumatic morbidity Australian and New Zealand Journal of Psychiatry 1987 21 210 218 10.3109/00048678709160914 3675454

[B2] Norris FH Uhl GA Chronic stress as a mediator of acute stress: The case of Hurricane Hugo Journal of Applied Social Psychology 1993 23 16 1263 1284 10.1111/j.1559-1816.1993.tb01032.x

[B3] Catapano F Malafronte R Lepre F Cozzolino P Arnone R Lorenzo E Tartaglia G Starace F Magliano L Maj M Psychological consequences of the 1998 landslide in Sarno, Italy: A community study Acta Psychiatrica Scandinavica 2001 104 6 438 442 10.1034/j.1600-0447.2001.00512.x 11782236

[B4] Fullerton CS Ursano RJ Kao TC Bharitya VR Disaster-related bereavement: Acute symptoms and subsequent depression Aviation, Space, and Environmental Medicine 1999 70 9 902 909 10503757

[B5] Wickrama KAS Wickrama KAT Family context of mental health risk in tsunami affected mothers: Findings from a pilot study in Sri Lanka Social Science & Medicine 2008 66 4 994 1007 10.1016/j.socscimed.2007.11.012 18164112

[B6] Schwarz ED Kowalski JM Malignant memories. Effect of a shooting in the workplace on school personnel's attitudes Journal of Interpersonal Violence 1993 8 4 468 485 10.1177/088626093008004003

[B7] Kaniasty K Norris FH Longitudinal linkages between perceived social support and posttraumatic stress symptoms: Sequential roles of social causation and social selection Journal of Traumatic Stress 2008 21 3 274 281 10.1002/jts.20334 18553415

[B8] Solomon SD Bravo M Rubio-Stipec M Canino G Effect of family role on response to disaster Journal of Traumatic Stress 1993 6 2 255 269 10.1002/jts.2490060208

[B9] Ursano RJ Fullerton CS Kao TC Bhartiya VR Longitudinal assessment of posttraumatic stress disorder and depression after exposure to traumatic death Journal of Nervous and Mental Disease 1995 183 1 36 42 10.1097/00005053-199501000-00007 7807068

[B10] Wahlström L Michelsen H Schulman A Backheden M Different types of exposure to the 2004 tsunami are associated with different levels of psychological distress and posttraumatic stress Journal of Traumatic Stress 2008 21 5 463 470 10.1002/jts.20360 18956445

[B11] Brooks N McKinlay W Mental health consequences of the Lockerbie disaster Journal of Traumatic Stress 1992 5 4 527 543 10.1002/jts.2490050403

[B12] Ranasinghe PD Levy BR Prevalence of and sex disparities in posttraumatic stress disorder in an internally displaced Sri Lankan population 6 months after the 2004 Tsunami Disaster Medicine and Public Health Preparedness 2007 1 1 34 41 10.1097/DMP.0b013e318068fbb7 18388601

[B13] Hollifield M Hewage C Gunawardena CN Kodituwakku P Bopagoda K Weerarathnege K Symptoms and coping in Sri Lanka 20-21 months after the 2004 tsunami British Journal of Psychiatry 2008 192 1 39 44 10.1192/bjp.bp.107.038422 18174508

[B14] Gleser GC Green BL Winget C Prolonged psychosocial effects of disaster: A study of Buffalo Creek 1981 New York, NY: Academic Press Inc

[B15] Kessler RC Sonnega A Bromet E Hughes M Nelson CB Posttraumatic stress disorder in the National Comorbidity Survey Archives of General Psychiatry 1995 52 12 1048 1060 10.1001/archpsyc.1995.03950240066012 7492257

[B16] Breslau N Peterson EL Poisson LM Schultz LR Lucia VC Estimating post-traumatic stress disorder in the community: Lifetime perspective and the impact of typical traumatic events Psychological Medicine 2004 34 5 889 898 10.1017/S0033291703001612 15500309

[B17] Weisæth L Arnetz BB, Ekman R Collective traumatic stress: Crisis and catastrophes Stress in health and disease 2006 Weinheim, Germany: WILEY-VCH Verlag GmbH & Co. KGaA

[B18] Lerner RM Concepts and theories of human development 1986 2 New York, NY: Newbery Award Records, Inc

[B19] Stern DN The interpersonal world of the infant. A view from psychoanalysis and developmental psychology 1985 New York, NY: Basic Books Inc

[B20] Siegel DJ The developing mind. Toward a neurobiology of interpersonal experience 1999 New York, NY: The Guilford Press

[B21] Berk LE Child development 2009 8 Boston, MA: Pearson

[B22] Bloch DA Silber E Perry SE Some factors in the emotional reaction of children to disaster American Journal of Psychiatry 1956 113 5 416 422 13362639 10.1176/ajp.113.5.416

[B23] McFarlane AC Posttraumatic phenomena in a longitudinal study of children following a natural disaster J Am Acad Child Adolesc Psychiatry 1987 26 5 764 769 10.1097/00004583-198709000-00025 3667509

[B24] Green BL Korol M Grace MC Vary MJ Leonard AC Gleser EC Smithson-Cohen S Children and disaster: Age, gender and parental effects on PTSD symptoms Journal of the American Academy of Child & Adolescent Psychiatry 1991 30 945 951 10.1097/00004583-199111000-00012 1757444

[B25] Catani C Jacob N Schauer E Kohila M Neuner F Family violence, war, and natural disasters: A study of the effect of extreme stress on children's mental health in Sri Lanka BMC Psychiatry 2008 8 33 42 10.1186/1471-244X-8-33 18454851 PMC2386780

[B26] Koplewicz HS Vogel JM Solanto MV Morrissey RF Alonso CM Abikoff H Gallagher R Novick RM Child and parent response to the 1993 World Trade Center bombing Journal of Traumatic Stress 2002 15 1 77 85 10.1023/A:1014339513128 11936725

[B27] Gershoff ET Aber JL Ware A Kotler JA Exposure to 9/11 among youth and their mothers in new york city: enduring associations with mental health and sociopolitical attitudes Child Dev 2010 81 4 1142 1160 10.1111/j.1467-8624.2010.01459.x 20636687 PMC7983060

[B28] Phillips D Featherman DL Jinyun L Children as an evocative influence on adults' reactions in terrorism Applied Developmental Science 2004 8 4 195 210 10.1207/s1532480xads0804_3

[B29] Kilmer RP Gil-Rivas V Responding to the needs of children and families after a disaster: Linkages between unmet needs and caregiver functioning American Journal of Orthopsychiatry 2010 80 1 135 142 10.1111/j.1939-0025.2010.01016.x 20397998 PMC2858329

[B30] Havenaar JM Rumyantzeva GM van den Brink W Poelijoe NW van den Bout J van Engeland H Koeter MW Long-term mental health effects of the Chernobyl disaster: An epidemiologic survey in two former Soviet regions American Journal of Psychiatry 1997 154 11 1605 1607 9356574 10.1176/ajp.154.11.1605

[B31] Stuber J Fairbrother G Galea S Pfefferbaum B Wilson-Genderson M Vlahov D Determinants of counseling for children in Manhattan after the September 11 attacks Psychiatric Services 2002 53 7 815 822 10.1176/appi.ps.53.7.815 12096163

[B32] Stuber J Resnick H Galea S Gender disparities in posttraumatic stress disorder after mass trauma Gender Medicine 2006 3 1 54 67 10.1016/S1550-8579(06)80194-4 16638601

[B33] Vila G Witkowski P Tondini MC Perez-Diaz F Mouren-Simeoni MC Jouvent R A study of posttraumatic disorders in children who experienced an industrial disaster in the Briey region European Child & Adolescent Psychiatry 2001 10 1 10 18 10.1007/s007870170042 11315531

[B34] Kristensen P Heir T Herlofsen PH Langsrud Ø Weisæth L Parental mental health after the accidental death of a son during military service: 23-year follow-up study Journal of Nervous and Mental Disease in press 10.1097/NMD.0b013e31823e5796 22210364

[B35] Asarnow J Glynn S Pynoos RS Nahum J Guthrie D Cantwell DP Franklin B When the earth stops shaking: Earthquake sequelae among children diagnosed for pre-earthquake psychopathology Journal of the American Academy of Child & Adolescent Psychiatry 1999 38 8 1016 1023 10.1097/00004583-199908000-00018 10434494

[B36] Nygaard E Jensen TK Dyb G Posttraumatic stress reactions in siblings after mutual disaster: Relevance of family factors Journal of Traumatic Stress 2010 23 2 278 281 20419737 10.1002/jts.20511

[B37] Goldstein H Multilevel mixed linear model analysis using iterative generalized least squares Biometrika 1986 73 1 43 56 10.1093/biomet/73.1.43

[B38] Norris FH Disaster research methods: Past progress and future directions Journal of Traumatic Stress 2006 19 2 173 184 10.1002/jts.20109 16612819

[B39] Dyb G Jensen TK Nygaard E Children's and parents' posttraumatic stress reactions after the 2004 tsunami Clinical Child Psychology and Psychiatry 2011 16 4 621 634 10.1177/1359104510391048 21565871

[B40] Nomura Y Chemtob CM Effect of maternal psychopathology on behavioral problems in preschool children exposed to terrorism: Use of generalized estimating equations to integrate multiple informant reports Archives of Pediatrics & Adolescent Medicin 2009 163 6 531 539 10.1001/archpediatrics.2009.51 19487609 PMC12965802

[B41] Heir T Weisæth L Acute disaster exposure and mental health complaints of Norwegian tsunami survivors six months post disaster Psychiatry 2008 71 3 266 276 10.1521/psyc.2008.71.3.266 18834277

[B42] Heir T Piatigorsky A Weisæth L Longitudinal changes in recalled perceived life threat after a natural disaster The British Journal of Psychiatry 2009 194 6 510 514 10.1192/bjp.bp.108.056580 19478289

[B43] Heir T Sandvik L Weisæth L Hallmarks of posttraumatic stress: Symptom Z-scores in a tsunami-affected tourist population Psychopathology 2009 42 3 157 164 10.1159/000207457 19276641

[B44] Hussain A Weisaeth L Heir T Nonresponse to a population-based postdisaster postal questionnaire study Journal of Traumatic Stress 2009 22 4 324 328 10.1002/jts.20431 19644976

[B45] Weisæth L Giller EL, Weisæth L The stressor-response relationship Post-traumatic stress disorder 1996 London: Bailliere Tindal 191 216

[B46] DSM-IV-TR Diagnostic and statistical manual of mental disorders 2000 4 Washington, DC: American Psychiatric Association text rev. edn

[B47] Weiss DS Marmar CR Wilson JP, Keane TM The Impact of Event Scale-Revised Assessing psychological trauma and PTSD 1997 New York, NY: Guilford Press 399 411

[B48] Weiss DS Wilson JP, Keane TM The Impact of Event Scale-Revised Assessing psychological trauma and PTSD 2004 New York, NY: The Guilford Press 168 189

[B49] Eid J Larsson G Johnsen BH Laberg JC Bartone PT Carlstedt B Psychometric properties of the Norwegian Impact of Event Scale-Revised in a non-clinical sample Nordic Journal of Psychiatry 2009 1 7 10.1080/08039480903118190 19688636

[B50] Schafer JL Analysis of incomplete multivariate data 1997 London, UK: Chapman & Hall

[B51] Efron B Tibshirani R Bootstrap methods for standard errors, confidence intervals, and other measures of statistical accuracy Statistical Science 1986 1 1 54 77 10.1214/ss/1177013815

[B52] Ozer EJ Best SR Lipsey TL Weiss DS Predictors of posttraumatic stress disorder and symptoms in adults: A meta-analysis Psychological Bulletin 2003 129 1 52 73 12555794 10.1037/0033-2909.129.1.52

[B53] Brewin CR Andrews B Valentine JD Meta-analysis of risk factors for posttraumatic stress disorder in trauma-exposed adults Journal of Consulting and Clinical Psychology 2000 68 5 748 766 11068961 10.1037//0022-006x.68.5.748

[B54] Sherif M The psychology of social norms 1936 New York, NY: Harper

[B55] Asch SE Social psychology 1952 Englewood Cliffs, NJ: Prentice Hall

[B56] Nichols MP Family therapy, concepts and methods 2010 9 Boston, MA: Pearson Education, Inc

[B57] Salmon K Bryant RA Posttaumatic stress disorder in children: The influence of developmental factors Clinical Psychology Review 2002 22 2 163 188 10.1016/S0272-7358(01)00086-1 11806018

[B58] Alisic E Jongmans MJ van Wesel F Kleber RJ Building child trauma theory from longitudinal studies: A meta-analysis Clinical Psychology Review 2011 31 5 736 747 10.1016/j.cpr.2011.03.001 21501581

[B59] Neuner F Schauer E Catani C Ruf M Elbert T Post-tsunami stress: A study of posttraumatic stress disorder in children living in three severely affected regions in Sri Lanka Journal of Traumatic Stress 2006 19 3 339 347 10.1002/jts.20121 16789000

